# Umbilical cord blood transplantation can overcome the poor prognosis of KMT2A-MLLT3 acute myeloid leukemia and can lead to good GVHD-free/relapse-free survival

**DOI:** 10.1007/s00277-021-04413-2

**Published:** 2021-01-20

**Authors:** Juan Tong, Lei Zhang, Huilan Liu, Xiucai Xu, Changcheng Zheng, Wen Yao, Xiaoyu Zhu, Baolin Tang, Xiang Wan, Kaidi Song, Xuhan Zhang, Guangyu Sun, Zimin Sun

**Affiliations:** Department of Hematology of Anhui Provincial Hospital, the First Affiliated Hospital of USTC, Division of Life Sciences and Medicine, University of Science and Technology of China, No. 17 Lujiang Road, Hefei, Anhui 230001 People’s Republic of China

**Keywords:** KMT2A-MLLT3, Cord blood transplant, Acute myeloid leukemia

## Abstract

This is a retrospective study comparing the effectiveness of umbilical cord blood transplantation (UCBT) and chemotherapy for patients in the first complete remission period for acute myeloid leukemia with KMT2A-MLLT3 rearrangements. A total of 22 patients were included, all of whom achieved first complete remission (CR1) through 1–2 rounds of induction chemotherapy, excluding patients with an early relapse. Twelve patients were treated with UCBT, and 10 patients were treated with chemotherapy after 2 to 4 courses of consolidation therapy. The 3-year overall survival (OS) of the UCBT group was 71.3% (95% CI, 34.4–89.8%), and that of the chemotherapy group was 10% (95% CI, 5.89–37.3%). The OS of the UCBT group was significantly higher than that of the chemotherapy group (*P* = 0.003). The disease-free survival (DFS) of the UCBT group was 60.8% (95% CI, 25.0–83.6%), which was significantly higher than the 10% (95% CI, 5.72–35.8%) of the chemotherapy group (*P* = 0.003). The relapse rate of the UCBT group was 23.6% (95% CI, 0–46.8%), and that of the chemotherapy group was 85.4% (95% CI, 35.8–98.4%), which was significantly higher than that of the UCBT group (*P* < 0.001). The non-relapse mortality (NRM) rate in the UCBT group was 19.8% (95% CI, 0–41.3%), and that in the chemotherapy group was 0.0%. The NRM rate in the UCBT group was higher than that in the chemotherapy group, but there was no significant difference between the two groups (*P* = 0.272). Two patients in the UCBT group relapsed, two died of acute and chronic GVHD, and one patient developed chronic GVHD 140 days after UCBT and is still alive, so the GVHD-free/relapse-free survival (GRFS) was 50% (95% CI, 17.2–76.1%). AML patients with KMT2A-MLLT3 rearrangements who receive chemotherapy as their consolidation therapy after CR1 have a very poor prognosis. UCBT can overcome the poor prognosis and significantly improve survival, and the GRFS for these patients is very good. We suggest that UCBT is a better choice than chemotherapy for KMT2A-MLLT3 patients.

## Introduction

In 2016, the WHO identified the mixed-lineage leukemia (MLL) gene KMT2A (lysine (K)-specific methyltransferase 2A), encoded in the 11q23 chromosomal region [1]. It can have different partner chromosomes/partner genes, and at present, more than sixty partner chromosomes/partner genes have been found. Among the recurrent 11q23/KMT2A rearrangements in acute myeloid leukemia (AML), the most common is t(9;11)(p22;q23), which results in a fusion of KMT2A with the MLLT3 gene (KMT2A-MLLT3, previously known as MLL/AF9). The incidence of AML with KMT2A-MLLT3 rearrangement is approximately 5% in adults and close to 15% in pediatric AML patients [2]. Most AML patients with 11q23/KMT2A rearrangements have worse outcomes, but KMT2A-MLLT3 is controversial. Risk stratification by the European LeukemiaNet classifies KMT2A-MLLT3 into moderate risk, while other partner gene translocations are considered high risk. However, there are also some studies, including some large-scale reports, that are different from the above conclusion, suggesting that KMT2A-MLLT3 is also a high-risk gene and has a poor prognosis [3].

According to the recommendations of the NCCN guidelines, it is still uncertain which treatment is the most suitable for AML patients at medium risk. Therefore, KMT2A-MLLT3 patients can choose either hematopoietic stem cell transplantation (HSCT) or chemotherapy for postremission treatment according to the risk stratification of genetics of the European LeukemiaNet for AML. According to the latest research of Huang XJ [4], the disease-free survival rate of patients with middle-risk AML who choose HSCT for postremission consolidation treatment is significantly better than those who choose chemotherapy. At present, there is no relevant literature about the difference in the final efficacy of KMT2A-MLLT3 between the two treatments of HSCT and chemotherapy.

Because of China’s national conditions, there are very few HLA-compatible donors at present. Umbilical cord blood transplantation (UCBT) is an important approach for patients with hematological diseases, and its use has made HSCT available to additional patients [5]. Starting in 2007, our transplant center began to use an intensive myeloablative conditioning regimen without antithymocyte globulin (ATG) for UCBT and cyclosporine combined with mycophenolate mofetil for graft-versus-host disease (GVHD) prophylaxis [6, 7]. After using this protocol, we increased the UCBT engraftment rate; in addition, the infection rate, transplantation-related mortality, and relapse rate were decreased, while the incidence of acute GVHD (aGVHD) and chronic GVHD (cGVHD) did not increase [8]. In a multicenter study in China, we observed similar survival when comparing UCBT without ATG and unrelated peripheral blood stem cell transplantation (UPBSCT) but better quality of life in patients undergoing UCBT without ATG [9]. Therefore, we chose UCBT to treat patients with acute leukemia when an HLA-matched sibling donor was not available.

In this study, we compared the efficacy of UCBT and conventional chemotherapy in AML patients with KMT2A-MLLT3 gene mutations for the first time. There were 22 patients with KMT2A-MLLT3 gene mutations, 12 of whom chose UCBT as postremission treatment after 2–3 high-dose cytarabine consolidation treatments, and 10 of whom chose to continue to receive chemotherapy as further treatment after high-dose cytarabine consolidation treatment. We analyzed and compared the data of the two groups.

## Patients and methods

### Patient

Between October 2010 and December 2018, 22 consecutive patients with acute myeloid leukemia with KMT2A-MLLT3 rearrangements were included in the study. To increase the comparability of the data, all patients were selected for 1–2 courses of treatment to achieve a complete remission (CR), all included patients were in their first complete remission period (CR1) when they selected their treatment, and patients with an early relapse (relapse within half a year after the first remission period) were excluded. There are some criteria for patients (and their parents for pediatric patients) to select the chemotherapy or UCBT groups. First, patients without suitable HSCT donors will enter the chemotherapy group. Second, patients with suitable UCBT donors will also enter the chemotherapy group if the patients are unwilling to undergo HSCT. Finally, patients who had suitable UCBT donors and agreed to receive UCBT were enrolled in the UCBT group. Finally, twelve patients chose UCBT, and 10 patients chose chemotherapy. The patient characteristics are shown in Table 1. All of the patients and their parents (pediatric patients) were fully informed of their disease status and their treatment options. The treatment protocol was approved by the University of Science and Technology of China Institutional Review Board.

**Table 1:** Patient characteristics

	UCBT group (12)	Chemotherapy group (10)	*P*
Sex (male, %)	6 (50%)	5 (50%)	1.0
Age	9.5 (2~59)	8.5 (2~37)	0.711
Morphology			0.854
M5	7	7	
M4	3	2	
M2	2	1	
Other combined chromosomal changes	1 (8.3%)	1 (10%)	0.795
Abnormal chromosome	46,XY,t(9;11)(p21;q23),+19	46,XY,t(9;11)(p21;q23),+8,del(8;12)(q10;q10)	
Number of leukocytes at diagnosis	31.4 (1.0–140.0)	39 (1.39–108)	0.872
Time to achieve CR	42 (24–68) days	35 (22–63) days	0.262

*UCBT*, umbilical cord blood transplantation; *CR*, complete remission

### Detection of KMT2A-MLLT3

Quantitative detection of the KMT2A-MLLT3 gene is needed in patients at disease onset and during residual disease monitoring. KMT2A-MLLT3 was measured once a month in the first four courses of treatment and then every two months. Bone marrow PBMCs were purified by density centrifugation using a standard Ficoll-Hypaque method. Total RNA was isolated from the bone marrow PBMCs and reverse transcribed to complementary DNA (cDNA). The qualitative detection of KMT2A-MLLT3 was performed as previously described [10].

### Diagnosis and treatment process

AML was diagnosed according to the WHO 2016 criteria and as described previously [1, 11]. Cytogenetic studies were carried out using standard techniques. Molecular screening of fusion genes was offered to all patients. The IA regimen (idarubicin 10–12 mg/m2 d 1-3 + cytarabine 100 mg/m2 d 1-7) was selected for the first round of induction chemotherapy. If the first round of induction chemotherapy did not achieve a CR, HA (homoharringtonine 2 mg/m^2^ d 1~7+ cytarabine 100 mg/m^2^ d 1-7), FLAG (fludarabine 30 mg/m^2^ d1~5+ cytarabine 2 g/m^2^ d 1-5 + G-CSF 5 μg/kg d 0-5) or CAG (aclacinomycin 20 mg/d d 1~4 + cytarabine 10 mg/m2 q 12 h d 1-14 + G-CSF 5 μg/kg d 1-14) regimens can be used as reinduction chemotherapy according to the condition of the myelodysplasia. After remission, a high dose of cytarabine (2–3 g/m2, every 12 h, D1-3) was given to all patients as consolidation treatment for 2–3 courses. The patients in the transplantation group received UCBT after consolidation treatment. The patients in the chemotherapy group were given IA, HA, MA (mitoxantrone 5 mg/m^2^ D1-3 + cytarabine 100 mg/m^2^ D1-7) and EA (VP-16 100 mg/m^2^ D1-3 + cytarabine 100 mg/m^2^ D1-7) alternately for 5 to 6 courses as maintenance therapy after consolidation chemotherapy.

### HLA typing and cord blood selection

High-resolution DNA typing for HLA-A, -B, -C, -DRB1, and –DQ was performed. The HLA-A, HLA-B and HLA-DRB1 antigens were typed using standard serological techniques, and HLA-C and DQ alleles were typed using high-resolution DNA techniques. Cord blood units were found through the Chinese Cord Blood Bank Network. Cord blood units matched at 4 or more of 6 HLA loci were selected. Preferred cord blood units contained a minimal cell count of 3 × 10^7^ nucleated cells/kg and 1.5 × 10^5^ CD34-positive cells/kg before freezing. Additionally, ABO compatibility was also considered. That is, the first choice is a matched blood type between the cord blood and the patient, and the second choice is a minor incompatibility blood type. However, if there is no matched or minor incompatibility blood type of cord blood available, cord blood with the major incompatibility and the primary and secondary incompatibility blood types can also be chosen. All of the CB units tested negative for human immunodeficiency virus (HIV), hepatitis B and C viruses (HBV, HCV) and human T cell lymphotropic virus type I. All of the CB units and mothers were negative for the immunoglobulin M antibody to cytomegalovirus (CMV). All of the CB units were transfused into the central venous blood.

### Conditioning regimen and GVHD prophylaxis

The myeloablative conditioning regimen for UCBT was based on classic BU/CY_2_ and TBI/CY_2_. Because TBI may affect the growth of the patients, only patients with central nervous system leukemia who were taller than 1.6 m were treated with TBI/CY_2_/Ara-c (TBI 3GY BID d-7 and d-6; Ara-c 2 g/m^2^ q 12 h d-5 and d-4; CY 60 mg/kg d-3 and d-2), the other patients were treated with BU/CY_2_ plus FLU (30 mg/m^2^ days -8 to -5; BU 0.8 mg/kg q6 h days -7 to -4; CY 60 mg/kg d-3 and d-2). The GVHD prophylaxis regimens were cyclosporine A and mycophenolate mofetil for all patients.

### Definitions and statistical analysis

CR was defined as less than 5% bone marrow (BM) blasts, absence of blasts with Auer rods, absence of extramedullary disease, 1.0 × 10^9^/L absolute neutrophil count, and 100 × 10^9^/L platelet (PLT) count. Minimal residual disease (MRD) was defined as previously reported [12]: > 0.01% flow cytometry MRD-positive (FCM MRD+) cells from a leukemia-associated immunophenotype (LAIP) cell population. Relapse was defined by the morphological evidence of disease in the peripheral blood, bone marrow, or extramedullary sites, and time to relapse was defined as the number of days from CR1 to the first diagnosis of relapse. Disease-free survival (DFS) was the primary endpoint, which was defined as the survival period with a continuous CR from CR1 after induction. MRD was not considered a relapse for DFS determination. Overall survival (OS) was defined as the number of days from the time of AML onset to death from any cause. Non-relapse mortality (NRM) was defined as death from any cause other than recurrent malignancy, and time to NRM was defined as the number of days from transplantation to death without a preceding relapse.

Primary graft failure was defined as profound, persistent pancytopenia and marrow hypoplasia without donor-derived cells on day 28 or reconstitution with autologous cells. Neutrophil engraftment was defined as the first of 3 consecutive days with an absolute neutrophil count of 0.5 × 10^9^/L, and platelet engraftment was defined as the first day when the platelet count was 20 × 10^9^/L for 7 consecutive days without transfusion support. Both aGVHD and cGVHD were diagnosed and graded according to the National Institutes of Health (NIH) consensus criteria [12, 13]. The novel composite end-point of GVHD-free/relapse-free survival (GRFS) after HCT is defined as patients without grade III-IV aGVHD, cGVHD requiring systemic treatment, relapse, or death [14].

Variables of the 2 groups were compared via the chi-square test (categorical covariates) or Mann-Whitney U-test (continuous covariates). Time-to-event outcomes for neutrophil and platelet engraftment, GVHD, NRM, and relapse were estimated using cumulative incidence curves, and these analyses were performed with R statistical software (R software 2.15) because of the presence of competing risks. For neutrophil or platelet engraftment and GVHD, death without an event was the competing risk; for NRM, relapse was the competing event, and for relapse, NRM was the competing event. The probabilities of OS and DFS were calculated using the Kaplan-Meier method, and these analyses were performed with SPSS (version 22.0). Differences at *P* < 0.05 were considered significant.

## Results

### Disease-free survival

**Fig. 1:**
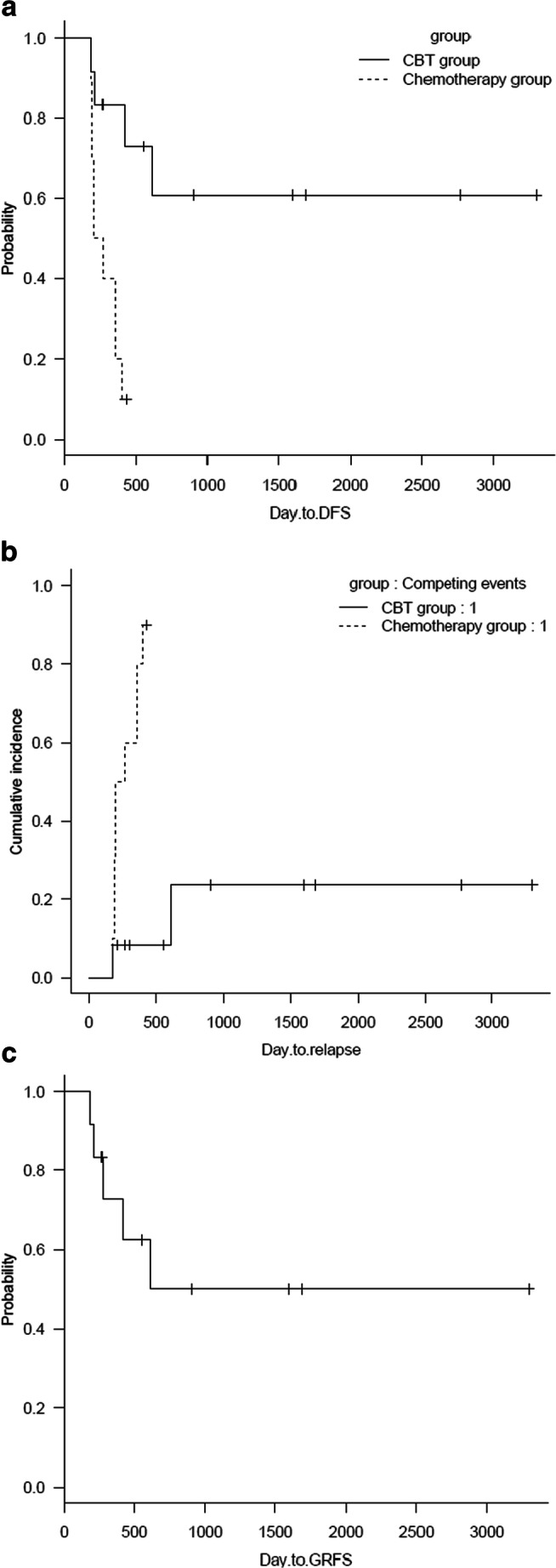
**a** The 3-year DFS was 60.8% in the UCBT group and 10% in the chemotherapy group, and the 3-year DFS in the UCBT group was significantly higher than that in the chemotherapy group (*P* = 0.003). **b** The relapse rate was 23.6% in the UCBT group and 85.4% in the chemotherapy group, and the relapse rate in the UCBT group was significantly lower than that in the chemotherapy group (*P* < 0.001). **c** The 3-year GRFs was 50% in the UCBT group

By the end of follow-up (August 1, 2020), in the chemotherapy group, 9 patients relapsed, and all relapsed patients died. The 3-year DFS was 10% (95% CI, 5.72–35.8%). Only 2 patients relapsed, and the DFS was 60.8% (95% CI, 25.0–83.6%) in the UCBT group, which was significantly better than that in the chemotherapy group (*P* = 0.003) (Fig. 1a).

### Overall survival

The 3-year overall survival (OS) of the UCBT group was 71.3% (95% CI, 34.4–89.8%). The 3-year OS of the chemotherapy group was 10% (95% CI, 5.89–37.3%). The total survival rate of the UCBT group was significantly better than that of the chemotherapy group (*P* = 0.003). The median survival of the chemotherapy group was 270 days.

### Minimal residual disease and relapse

KMT2A-MLLT3 was negative in all patients with complete remission of acute leukemia but positive in all patients when they relapsed. The relapse rate was 23.6% (95% CI, 0–46.8%) in the UCBT group and 85.4% (95% CI, 35.8–98.4%) in the chemotherapy group. The relapse rate in the chemotherapy group was significantly higher than that in the UCBT group (*P* < 0.001). The relapse times of the two patients in the UCBT group were 185 days and 611 days, respectively. The median relapse time in the chemotherapy group was 200 days (187–400 days). In the UCBT group, one patient died of relapse, and one patient with central nervous system leukemia is still alive after intrathecal chemotherapy and radiotherapy (Fig. 1b).

### Non-relapse mortality

Two patients in the UCBT group died of acute GVHD and chronic GVHD. The incidence of NRM was 19.8% (95% CI, 0–41.3%), while the incidence of NRM was 0.0% in the chemotherapy group. The incidence of NRM in the UCBT group was higher than that in the chemotherapy group, but there was no significant difference between the two groups (*P* = 0.272).

### GVHD-free/relapse-free survival in the UCBT group

The total nucleated cell dose that UCBT patients received was 5.4 (2.3–9.6) ×10^7^/kg and the CD34^+^ cell dose was 2.3 (1.5–5.2) ×10^5^/kg. All UCBT patients successfully achieved neutrophil engraftment on 17 (12-31) days and platelet engraftment on 39 (17–46) days. Two patients in the UCBT group relapsed, two died of acute and chronic GVHD, and one patient among the surviving patients developed chronic GVHD at 140 days after UCBT. The NIH score of chronic GVHD was 2. GRFS was 50% (95% CI, 17.2–76.1%) (Fig. 1c).

## Discussion

In 1986, scientists first used cell morphology, immunophenotype, and cytogenetics to classify AML and generated the M5a/t (11q) classification at the same time. By 2001, WHO updated the classification method of AML, one of which was AML with recurrent gene abnormalities, including AML with 11q23/MLL (mixed lineage leukemia) rearrangement abnormalities.

In a study involving 1897 AML patients, the morphological characteristics of bone marrow cells of 11q23/MLL AML patients were analyzed [15]. The proportions of M4, M5a, and M5b corresponding to French American British (FAB) typing and MLL mutation patients were 4.7%, 33.3%, and 15.9%, respectively. They were found in only 0.9% of all other FAB subtypes (*P* < 0.0001). Since 2016, WHO has named the 11q23/MLL gene 11q23/KMT2A, and KMT2A-MLLT3 (MLL-AF9) is one of the many KMT2A mutations, which is a gene mutation with controversy over its effects. In the early stages of acute leukemia genotyping, most studies found that cases with KMT2A-MLLT3 had a better prognosis, which was related to an increased sensitivity to chemotherapy drugs. Palle J et al. [16] found that in AML, children with t(9;11) (*n* = 10) were significantly more sensitive to cytarabine (*P* < 0·001) and doxorubicin (*P* = 0·005) than non-11q23 rearranged patients (*n* = 108). Children with other 11q23 rearrangements (*n* = 14) differed less from non-rearranged children. It is not known why infants fare much worse than older children with the same genetic subtypes of ALL and why t(9;11) has a favorable prognostic impact in AML, while other 11q23 rearrangements do not. However, in vitro studies of cellular drug resistance have indicated that drug resistance at the cellular level might be one factor of importance.

Zwaan CM et al. [17] found that t(9;11) samples from children with AML were more sensitive to a number of drugs than the other AML samples, and Ramakers-van Woerden et al. [18] reported significant differences in the drug sensitivity profile of MLL-rearranged samples from children with ALL compared with non-MLL-rearranged samples. Both of these studies were comprised of patients treated with a number of different protocols, and no direct comparisons between ALL and AML were made.

However, over time, many studies found that KMT2A-MLLT3 was not associated with a good prognosis. In a study of 756 pediatric patients, Balgobind BV et al. [3] found that t (1; 11) (q21; q23) and t (10; 11) (p12; q23) were independent factors associated with a poor prognosis, KMT2A-MLLT3 was not in a group with a good prognosis, and there was no significant difference from the other MLL rearrangements.

In 2008, eight adult patients with AML with the 11q23 mutation were treated with UCBT for the first time in Japan [19]. Among them, one patient failed to achieve engraftment, and the median time of granulocyte engraftment was 22 days. Seven patients developed aGVHD, and 6 patients developed cGVHD. Transplantation-related mortality was observed for one patient on day 49 (graft failure). Three patients experienced a relapse, two of whom died of the relapse, while the remaining patient survived. Four patients remained alive free of disease between 182 and 1074 days after transplantation. With a median follow-up of 425 days, the estimated DFS at 1 year was 58%. Although the patient cohort was small and the observation period was short, the authors’ experience suggests that it is appropriate to consider myeloablative CBT early in the course for adult AML patients with 11q23 abnormalities if a matched related donor is not available.

Because there was no previous study reporting the results of a large number of AML cases with 11q23 mutation patients treated with HSCT, Pigneux A et al [20] reported the effectiveness of hematopoietic stem cell transplantation for 159 adult patients with 11q23/KMT2A rearranged AML in their first complete remission (CR1, n = 138) or CR2 in 2015. Half of them received stem cells from matched sibling donors, and the other half received stem cells from unrelated donors. Sixty-five percent of the patients received myeloablative transplantation, and the rest received nonmyeloablative transplantation. Most patients had t(9;11), t(11;19), t(6;11), and t(10;11) translocations. The treatment was more favorable in patients with t(9;11) and t(11;19) than in patients with t(10;11) and t(6;11) (2-year OS: 64 ± 6% and 73 ± 10% vs. 40 ± 13% and 24 ± 11%, respectively). This study showed that HSCT could improve the prognosis of MLL rearrangement patients, especially KMT2A-MLLT3 rearrangement patients.

Our research reached the same conclusion. We found that the prognosis of AML patients with KMT2A-MLLT3-rearrangements treated with conventional chemotherapy was very poor; they had a very high relapse rate, and few patients achieved long-term survival. In the group that was treated with UCBT, DFS and OS were significantly improved for these KMT2A-MLLT3 patients, and the patients also had better GRFS, indicating that the quality of life of these patients was very good. For the conventional chemotherapy group, the OS of the patients was significantly worse than that in previous reports, which may be related to their different races. We will carry out further research to explore the reasons for this discrepancy.

In conclusion, our findings suggest that the prognosis of KMT2A-MLLT3 AML patients treated with conventional chemotherapy is very poor, and it is recommended to use UCBT for treatment. Looking for drugs to target KMT2A-MLLT3 is our future treatment direction. Of course, there are some limitations of this study. First, the number of cases is small. Second, this is a retrospective study, not a prospective randomized controlled study.
